# Multianalytical Characterization of Elderberry (*Sambucus nigra* L.) Pulp-Enriched Kefir Ice Cream: From Structure to Aroma and Sensory Quality

**DOI:** 10.3390/foods15173025

**Published:** 2026-08-27

**Authors:** Duygu Güçyetmez, Melike Demirkol, Ömer Faruk Çelik

**Affiliations:** 1 Department of Food Engineering, Faculty of Engineering and Architecture, Tokat Gaziosmanpaşa University, 60250 Tokat, Türkiye; gucyetmezduygu0020@gmail.com; 2 Department of Food Engineering, Faculty of Agriculture, Ordu University, 52200 Ordu, Türkiye; ofarukcelik@gmail.com

**Keywords:** functional dairy products, frozen fermented dairy products, phenolic compounds, volatile profile, thermal properties, microstructure

## Abstract

The development of functional frozen dairy products enriched with natural bioactive ingredients has attracted increasing interest. Elderberry (*Sambucus nigra* L.) is an anthocyanin-rich fruit with potential as a natural functional and coloring ingredient in dairy products. This study investigated the effects of incorporating 3%, 6%, and 9% elderberry pulp into kefir ice cream on its physicochemical, antioxidant, thermal, microstructural, volatile, and sensory properties. The study provides an integrated evaluation of elderberry-enriched kefir ice cream by combining physicochemical and antioxidant characterization with DSC, SEM, HS-SPME-GC/MS, and sensory analyses. Elderberry supplementation significantly decreased pH, increased redness, and enhanced total phenolic content and ferric reducing antioxidant power, which reached 694.16 mg GAE/100 g DM and 49.70 µmol TE/100 g DM, respectively, in EP9 (*p* < 0.05). Formulations containing elderberry pulp also showed significant differences in overrun, density, melting behaviour, and thermal properties (*p* < 0.05). Differential scanning calorimetry indicated lower melting enthalpy values in the elderberry-containing formulations, while scanning electron microscopy revealed modifications in porous microstructure. HS-SPME-GC/MS analysis tentatively identified 25 volatile compounds, with ketones representing the predominant volatile group and elderberry supplementation modifying the relative abundance of selected volatile compounds. Sensory evaluation showed improved appearance, flavor, texture, and overall acceptability compared with the kefir control (*p* < 0.05). Overall, formulations containing 6% and 9% elderberry pulp provided a favorable balance between technological quality, functional potential, and sensory acceptability, suggesting a practical formulation range for the development of naturally colored, functional kefir-based frozen dairy products.

## 1. Introduction

The growing recognition of the relationship between diet and health has shifted consumer preferences from conventional foods toward products that provide physiological benefits beyond basic nutrition, thereby accelerating the development of functional foods worldwide [1,2]. Functional dairy products, particularly frozen dairy desserts, have attracted increasing scientific and industrial interest because they represent an effective matrix for delivering health-promoting ingredients while maintaining high consumer acceptance and desirable sensory properties [3,4]. In parallel with the increasing demand for natural ingredients, considerable research has focused on incorporating naturally derived ingredients that can simultaneously improve nutritional value, technological functionality, and product stability [1,2,5]. Consequently, a wide range of plant-derived proteins, dietary fibers, fruit materials, and phytochemical-rich ingredients have been incorporated into ice cream formulations to enhance antioxidant capacity, improve physicochemical and rheological properties, optimize melting behavior, and increase consumer acceptability without compromising product quality [4,6,7]. These advances have positioned functional frozen dairy products as one of the fastest-growing sectors of the dairy industry, creating opportunities for the development of innovative formulations enriched with natural bioactive compounds [1,3]. Among fermented dairy products, kefir is distinguished by its highly diverse microbial consortium, in which lactic acid bacteria, acetic acid bacteria, and yeasts coexist within a symbiotic matrix known as kefir grains [8,9]. During fermentation, microbial metabolism leads to the formation of numerous bioactive constituents, including organic acids, exopolysaccharides, bioactive peptides, vitamins, and volatile compounds that collectively determine the nutritional, technological, and sensory characteristics of the final product [9,10]. These metabolites have been associated with a wide range of biological activities, such as antioxidant, antimicrobial, immunomodulatory, anti-inflammatory, and antihypertensive effects, reinforcing kefir’s status as one of the most promising functional fermented dairy products [8,11]. Beyond its health-promoting potential, kefir has increasingly been considered a versatile fermentation matrix for the development of innovative dairy products, as recent studies have demonstrated that the incorporation of functional ingredients and emerging processing technologies can improve both product stability and consumer acceptability while preserving its biological functionality [12].

The incorporation of kefir into ice cream has recently emerged as a promising strategy to combine the nutritional and functional advantages of fermented dairy products with the widespread consumer acceptance of frozen desserts [12]. Compared with conventional ice cream, kefir-based ice cream offers the potential to deliver microbial metabolites, bioactive compounds, and fermentation-derived flavor components within a product matrix that is generally perceived as more appealing by consumers [8,12]. In addition to improving the functional value of the final product, kefir fermentation may also influence the physicochemical and microstructural characteristics of ice cream through modifications in protein interactions, water distribution, and exopolysaccharide production, which are closely associated with texture, melting behavior, and overall product stability [1,7]. Consequently, increasing attention has been directed toward enriching kefir-based frozen dairy products with plant-derived ingredients rich in phenolic compounds and natural pigments to further enhance their nutritional quality and technological performance while meeting consumer demand for natural and health-oriented foods [4].

Berry fruits have attracted considerable attention as natural functional ingredients because of their high concentrations of phenolic compounds, particularly anthocyanins, flavonoids, and phenolic acids, which exhibit strong antioxidant, anti-inflammatory, antimicrobial, and cardioprotective activities [13,14]. Among these fruits, elderberry (*Sambucus nigra* L.) is recognized as one of the richest natural sources of anthocyanins, with cyanidin-based glycosides representing the predominant pigments responsible for its characteristic dark purple color and remarkable antioxidant capacity. In addition to anthocyanins, elderberry contains substantial amounts of phenolic acids, flavonols, vitamins, minerals, and other phytochemicals that collectively contribute to its nutritional value and biological activities [13,15]. Elderberry fruits also contain appreciable amounts of dietary fiber, including pectic substances such as pectin, pectic acid, protopectin, and calcium pectate, which further contribute to their potential as functional food ingredients [16]. Consequently, elderberry has been increasingly incorporated into various food systems, including jelly and juice [17], yogurt [18], and kefir [19], to improve their functional properties, enhance natural coloration, and increase the content of health-promoting phytochemicals without the use of synthetic additives [13,19]. Although elderberry has demonstrated considerable potential as a functional ingredient in dairy products, existing studies have predominantly focused on yogurt and fermented beverages. Consequently, its application in kefir-based frozen dairy products remains largely unexplored, particularly regarding product quality and structural stability. Moreover, available studies have generally emphasized basic physicochemical and antioxidant properties, whereas the relationships among technological properties, thermal behavior, microstructural organization, volatile composition, and sensory characteristics within a fermented frozen dairy matrix remain insufficiently understood. This represents an important knowledge gap because the incorporation of fruit pulp into kefir ice cream may simultaneously affect the aqueous, protein, fat, and air phases that collectively determine product structure and quality. Accordingly, it was hypothesized that elderberry pulp incorporation would enhance the functional and sensory properties of kefir ice cream while also influencing its physicochemical, thermal, microstructural, and volatile characteristics.

Therefore, the present study aimed to develop kefir ice cream enriched with different concentrations of elderberry (*Sambucus nigra* L.) pulp and to comprehensively evaluate its physicochemical, textural, bioactive, thermal, microstructural, volatile, and sensory properties. Unlike studies focusing on individual quality attributes, the combined use of DSC, SEM, and HS-SPME-GC/MS alongside conventional physicochemical, antioxidant, and sensory analyses enabled complementary evaluation of thermal transitions, matrix organization, and changes in the volatile profile. This integrated approach was intended to provide a more comprehensive understanding of how elderberry pulp incorporation influences the technological and sensory characteristics of kefir ice cream and to clarify its potential as a natural functional ingredient in fermented frozen dairy products.

## 2. Materials and Methods

### 2.1. Preparation and Characterization of Elderberry Pulp

Ripe elderberry (*Sambucus nigra* L.) fruits (Haschberg cultivar) were obtained from the Central Black Sea Transitional Zone Agricultural Research Institute (Tokat, Türkiye) during the September–October 2025 harvest season. The fruits were transported to the laboratory under refrigerated conditions and stored at −18 °C until use. The frozen fruits were thawed prior to pulp preparation, sorted to remove stems, leaves, and other foreign materials and then thoroughly washed with cold water. The cleaned fruits were homogenized using a laboratory blender (HGB2WTS3, Waring Commercial, Torrington, CT, USA). The resulting homogenate was subsequently passed through a fine sieve, and the seeds and coarse skin fragments retained on the sieve were discarded, while the edible fruit tissue passing through the sieve was collected as elderberry pulp. Before establishing the final processing procedure, total phenolic content and antioxidant activity were assessed before and after pasteurization, with no appreciable differences observed between the two conditions. Accordingly, the pulp was pasteurized at 85 °C for 15 min, cooled to room temperature, and subsequently used for physicochemical analyses and kefir ice cream manufacture.

The pH of the elderberry pulp was measured using a digital pH meter (HI 2211, Hanna Instruments, Woonsocket, RI, USA). Titratable acidity was determined by diluting 10 g of pulp with 50 mL of distilled water, followed by titration with 0.1 N of NaOH (Merck, Darmstadt, Germany) to an endpoint of pH 8.1. Titratable acidity was calculated according to the following equation:

Titratable acidity (%) = *V* × *N* × 0.06404 × 100/*m* (1)

where *V* is the volume of NaOH (mL), *N* is the normality of NaOH, 0.06404 is the milliequivalent factor for citric acid, and *m* is the sample weight (g). The results were expressed as percentage of citric acid on a fresh weight (FW) basis. Moisture, total solids, ash, and protein contents were determined according to the standard methods of the AOAC [20].

For the determination of bioactive properties, phenolic compounds were extracted according to the method of Veberic et al. [21] with minor modifications. Briefly, 2 g of elderberry pulp was mixed with 40 mL of acidified methanol (HCl/methanol (Sigma-Aldrich, St. Louis, MO, USA)/water, 1:80:19, *v*/*v*/*v*) and extracted in an ultrasonic water bath (45 kHz) (TI-H-15, Elma Schmidbauer GmbH, Singen, Germany) at 25 °C for 30 min using 80% of the maximum ultrasonic power. The extracts were centrifuged at 6000 rpm for 10 min at 4 °C, and the resulting supernatants were collected and stored at −18 °C until analysis. Total phenolic content (TPC) was determined using the Folin–Ciocalteu reagent (Sigma-Aldrich, St. Louis, MO, USA) according to the method described by Singleton and Rossi Jr [22]. Results were calculated from the gallic acid (Sigma-Aldrich, St. Louis, MO, USA) calibration curve and expressed as mg gallic acid equivalents (GAE) per gram of fresh weight (mg GAE/g FW). Ferric reducing antioxidant power (FRAP) was determined according to the procedure described by Benzie and Strain [20], and the results were expressed as µmol Trolox equivalents (TE) per gram of fresh weight (µmol TE/g FW). DPPH radical scavenging activity was determined based on the method described by Brand-Williams et al. [23], with modifications. Briefly, 50 µL of the appropriately diluted extract was mixed with 1 mL of DPPH (2,2-diphenyl-1-picrylhydrazyl; Sigma-Aldrich, St. Louis, MO, USA) solution prepared in methanol (10^−3^ mol/L). The mixture was incubated in the dark for 30 min, and absorbance was measured at 515 nm. Antioxidant capacity was calculated using a Trolox (Sigma-Aldrich, St. Louis, MO, USA) calibration curve and expressed as mg Trolox equivalents (TE) per gram of fresh weight (mg TE/g FW).

### 2.2. Preparation of Kefir

Commercial pasteurized cow’s milk (3.1% fat, 3.0% protein, and 4.7% carbohydrates; Güney Süt San. ve Gıda Mad. Tic. A.Ş., Mersin, Türkiye) was used for kefir production. Prior to inoculation, the milk was equilibrated to 25–30 °C and inoculated with a commercial direct-vat-set (DVS) kefir starter culture (eXact^®^ Kefir 1, Chr. Hansen, Hørsholm, Denmark) according to the manufacturer’s instructions. The starter culture contained *Lactococcus lactis* subsp. *lactis*, *Lactococcus lactis* subsp. *cremoris*, *Lactococcus lactis* subsp. *lactis* biovar *diacetylactis*, *Streptococcus thermophilus*, *Leuconostoc* spp., and *Debaryomyces hansenii*. The inoculated milk was incubated at 25–30 °C for 24 h to allow fermentation. Upon completion of fermentation, the kefir was stored at 4 ± 1 °C and used in the manufacture of kefir ice cream.

### 2.3. Preparation of Kefir Ice Cream

The ingredients used in the preparation of the kefir ice cream formulations were obtained from commercial suppliers. Pasteurized cow’s milk containing 3.1% fat (Güney Süt San. ve Gıda Mad. Tic. A.Ş., Mersin, Türkiye), cow’s milk cream containing 35% fat (Gönenli Süt ve Süt Ürünleri Gıda San. ve Tic. A.Ş., Balıkesir, Türkiye), and skim milk powder (Kozağaçlı Süt Çiftliği, Barbarossa Kimya Gıda San. Tic. Ltd. Şti., İstanbul, Türkiye) were used as the dairy ingredients. Granulated sugar (Orion Dış Tic. A.Ş., Konya, Türkiye) was used as the sweetener, while powdered salep (Saf Bucak Salebi, Aktarix Bitkisel Ürünler, Antalya, Türkiye) and mono- and diglycerides of fatty acids (E471; Pastaland, Ataygeldi Gıda San. ve Tic. Ltd. Şti., Bursa, Türkiye) were used as the stabilizer and emulsifier, respectively.

The formulations used for the production of kefir ice cream are presented in Table 1. Five experimental formulations were prepared, including a control ice cream without kefir and elderberry pulp (CO), a kefir control (KF), and kefir ice creams supplemented with 3% (EP3), 6% (EP6), and 9% (EP9) elderberry pulp. The concentrations of elderberry pulp (3%, 6%, and 9%, *w*/*w*) were selected based on preliminary formulation trials considering product stability, sensory acceptability, and technological suitability. During these preliminary trials, higher supplementation levels (>9%) were also evaluated; however, the simultaneous incorporation of kefir and higher amounts of elderberry pulp resulted in excessive acidity, an overly intense dark-purple color, and undesirable changes in texture and overall sensory quality. Therefore, the selected concentrations represented progressively increasing supplementation levels within the technologically and sensorially acceptable range, allowing the concentration-dependent effects of elderberry pulp to be systematically evaluated while maintaining acceptable product quality.

To evaluate the effect of elderberry pulp while minimizing changes in the overall formulation composition, the amounts of cream (8%), sucrose (15%), skim milk powder (3.2%), salep (0.4%), and emulsifier (0.4%; E471) were kept constant across all formulations, as shown in Table 1. Elderberry pulp was incorporated by proportionally reducing the amount of cow’s milk, while the kefir level was kept constant at 34%. The kefir level was determined based on the findings of Türkmen et al. [24] and preliminary sensory evaluations. The base formulations were developed based on the formulations reported by Ozturk-Yalcin et al. [25] and Januário et al. [26]. The ice cream mix was prepared using the ingredients according to the formulations in Table 1. The mixture was thoroughly homogenized and pasteurized at 80–85 °C for 25 s. After pasteurization, the mix was cooled to 4 °C and aged for 24 h to allow complete hydration of the dry ingredients and stabilization of the emulsion. Following the aging period, elderberry pulp was added to the formulations at the designated concentrations and thoroughly mixed. Kefir was subsequently incorporated into the respective formulations, and the mixes were gently blended to ensure uniform distribution of all ingredients. The mixes were then frozen in a batch ice cream freezer (M5, Mehen, Nanjing, China), packaged into 100 mL plastic containers, and stored at −18 °C until further analyses.

### 2.4. Physicochemical Analyses of Kefir Ice Cream

The pH and titratable acidity of the ice cream samples were determined after melting at room temperature. The pH was measured by directly immersing the electrode of a digital pH meter (HI 2211, Hanna Instruments, Woonsocket, RI, USA) into the homogenized samples. For titratable acidity, 9 g of melted ice cream was mixed with 9 mL of distilled water and titrated with 0.1 N of NaOH to pH 8.1. Titratable acidity was calculated using the following equation:

TA (% lactic acid) = *V* × *N* × 0.09 × 100/*m* (2)

where *V* is the volume of NaOH used (mL), *N* is the normality of NaOH, 0.09 is the conversion factor for lactic acid, and *m* is the sample weight (g). The results were expressed as percentage of lactic acid according to the method described by Bradley et al. [27]. Total solids, ash, protein, and fat contents were determined according to AOAC [20] official methods. Color measurements were performed on frozen ice cream samples using a spectrocolorimeter (DS-200, CHNSpec Technology Co., Ltd., Hangzhou, China). The color parameters were recorded according to the CIE L*, a*, and b* color system, where L* represents lightness, a* indicates redness (+) to greenness (−), and b* indicates yellowness (+) to blueness (−). The viscosity of the ice cream mixes was measured at 9 ± 1 °C using a Brookfield DV2T rotational viscometer (Brookfield Engineering Laboratories, Stoughton, MA, USA) equipped with an RV-03 spindle operating at 20 rpm. Viscosity values were recorded after 30 s and expressed as centipoise (cP). Overrun was determined from the weight difference between equal volumes of ice cream mix and the corresponding frozen product according to Muse and Hartel [28], and expressed as percentage. Ice cream density was determined using a container of known volume according to the method described by Khalil et al. [29], and the results were expressed as g/cm^3^. Melting behaviour was evaluated using 20 g of each ice cream sample placed on a wire mesh positioned above a beaker at room temperature. The time to first drip and the amount of melted ice cream collected after 30 min were recorded.

### 2.5. Determination of Bioactive Properties

Bioactive compounds were extracted from the ice cream samples using a procedure adapted from Beta et al. [30] with modifications. Briefly, 10 g of melted ice cream was mixed with 40 mL of acidified methanol (HCl/methanol/water, 1:80:19, *v*/*v*/*v*) and extracted in a shaking incubator (Ecotron, Infors AG, Bottmingen, Switzerland) at room temperature for 1 h at 100 rpm. Following extraction, the samples were centrifuged (MIKRO 220, Andreas Hettich GmbH & Co. KG, Tuttlingen, Germany) at 6000 rpm for 10 min at 4 °C, and the resulting supernatants were filtered prior to analysis. Total phenolic content (TPC) and ferric reducing antioxidant power (FRAP) were determined according to the analytical procedures described in Section 2.1. Pearson’s correlation coefficient (r) was used to evaluate the linear relationship between TPC and FRAP. The correlation coefficient was calculated using the replicate measurements, and statistical significance was evaluated at a significance level of *p* < 0.05. The results were calculated on a dry matter basis and expressed as mg GAE/100 g DM and µmol TE/100 g DM, respectively. DPPH analysis was also evaluated for the ice cream extracts; however, matrix-related turbidity prevented reliable spectrophotometric quantification. Therefore, antioxidant evaluation of the ice cream formulations was based on TPC and FRAP.

### 2.6. Texture Profile Analysis

The textural properties of the ice cream samples were determined using a TA.XT Plus texture analyzer (Stable Micro Systems Ltd., Godalming, UK) equipped with Exponent software (version 6.1.16.0). Measurements were performed in compression mode using a stainless-steel cylindrical probe (P/2, 2 mm diameter). The pre-test, test, and post-test speeds were set at 1.0, 1.0, and 5.0 mm/s, respectively. The penetration distance was 15 mm, and the trigger force was adjusted to 0.029 N. Prior to analysis, the ice cream samples were allowed to equilibrate to serving temperature and were placed in suitable containers to provide a uniform sample surface. Hardness (g) was recorded as the maximum positive force obtained during penetration, whereas stickiness (g) was calculated from the negative area of the force–time curve during probe withdrawal. Eight independent measurements were performed for each sample, and the results were expressed as mean ± standard deviation.

### 2.7. Differential Scanning Calorimetry (DSC)

The thermal properties of the ice cream samples were evaluated using a simultaneous differential scanning calorimeter (SDT Q600, TA Instruments, New Castle, DE, USA) following the method of Markowska et al. [31] with minor modifications. Approximately 5–10 mg of each sample was weighed into hermetically sealed aluminum pans, while an empty aluminum pan was used as the reference. Measurements were carried out under a nitrogen atmosphere.

The thermal program consisted of cooling the samples from 25 °C to −80 °C at a rate of 10 °C/min, followed by heating to −40 °C at the same rate and holding for 30 min. Subsequently, the samples were cooled again to −80 °C at 10 °C/min and equilibrated for 5 min. In the final step, the samples were heated from −80 °C to 20 °C at a rate of 5 °C/min. Onset, peak, and endset temperatures of the ice melting transition, together with the melting enthalpy (ΔH, J/g), were determined from the DSC thermograms using the using Universal Analysis 2000 software.

### 2.8. Scanning Electron Microscopy (SEM)

The microstructure of the ice cream samples was examined using a scanning electron microscope (SEM; SU1510, Hitachi, Tokyo, Japan) according to the method described by Yuliarti et al. [32]. Prior to SEM analysis, frozen ice cream samples were freeze-dried using a FreeZone 2.5 freeze dryer (Labconco, Kansas City, MO, USA) at −52 °C under a chamber pressure of 0.1 mbar for 48 h. The dried samples were carefully fractured into small pieces, mounted on aluminum stubs, and sputter-coated with a thin layer of gold to improve electrical conductivity. Micrographs were obtained at an accelerating voltage of 20 kV and a magnification of 1000×.

### 2.9. Analysis of Volatile Compounds by HS-SPME-GC/MS

Volatile compounds were determined by headspace solid-phase microextraction coupled with gas chromatography–mass spectrometry (HS-SPME-GC/MS) using a Shimadzu GCMS-QP2010 Ultra (Shimadzu Corporation, Kyoto, Japan) equipped with an RTX-5MS capillary column (30 m × 0.25 mm i.d., 0.25 µm film thickness). Approximately 5 g of each ice cream sample was placed in a headspace vial and equilibrated at 60 °C. Volatile compounds were extracted using a 50/30 µm DVB/CAR/PDMS fiber (Supelco, Bellefonte, PA, USA) for 20 min and thermally desorbed in the injector for 7 min. Helium was used as the carrier gas at a constant flow rate of 1.0 mL/min. Samples were injected in splitless mode at 200 °C. The oven temperature was programmed from 40 °C (2 min) to 200 °C at 4 °C/min (2 min hold), followed by heating to 230 °C at 10 °C/min with a final hold of 10 min. The mass spectrometer was operated with an ion source temperature of 200 °C and an interface temperature of 250 °C, and mass spectra were acquired over an m/z range of 35–400. Volatile compounds were tentatively identified by comparison of their mass spectra with those available in the FFNSC 3 and NIST 11 mass spectral libraries. Since retention indices and authentic reference standards were not used for confirmation, the compound assignments should be regarded as tentative.

### 2.10. Sensory Evaluation

Sensory evaluation was conducted in accordance with the Declaration of Helsinki and performed by 30 semi-trained panelists (aged 20–50 years) from the Department of Food Engineering, Tokat Gaziosmanpaşa University, who were familiar with kefir and ice cream products. Before the evaluation, the panelists were informed about the product characteristics, sensory attributes to be evaluated, and the scoring procedure. The sensory evaluation protocol was approved by the Tokat Gaziosmanpaşa University Ethics Committee for Research in Science and Engineering (Decision No. 02.06; Document No. 689471). Written informed consent was obtained from all participants prior to the study. Before evaluation, the ice cream samples were tempered to approximately −12 ± 2 °C and served in coded plastic cups under identical laboratory conditions. Random three-digit codes were assigned to the samples, and the presentation order was randomized for each panelist to minimize potential order effects. The evaluation was conducted in a laboratory environment arranged to minimize external distractions and interaction among panelists. Water was provided between samples to minimize carry-over effects. The panelists evaluated the samples for appearance, odor, flavor, melting in mouth, texture, graininess, and overall acceptability using a nine-point hedonic scale (1 = dislike extremely, 9 = like extremely), following the general sensory evaluation procedures described by Meilgaard et al. [33].

### 2.11. Statistical Analysis

The experiment was conducted in two independent production batches, which represented independent biological replicates, with each batch manufactured separately. The number of analytical replicate measurements for each analysis is specified in the corresponding table footnotes. Results were expressed as mean ± standard deviation. Statistical analyses were performed using SPSS Statistics 20.0 (IBM Corp., Armonk, NY, USA). Prior to ANOVA, the assumptions of normality and homogeneity of variance were assessed using the Shapiro–Wilk and Levene’s tests, respectively, and the assumptions were satisfied. Data were subjected to one-way analysis of variance (ANOVA), and significant differences among treatment means were determined using Tukey’s multiple comparison test. Differences were considered statistically significant at *p* < 0.05.

## 3. Results and Discussion

### 3.1. Characterization of Elderberry Pulp

**Figure 1:**
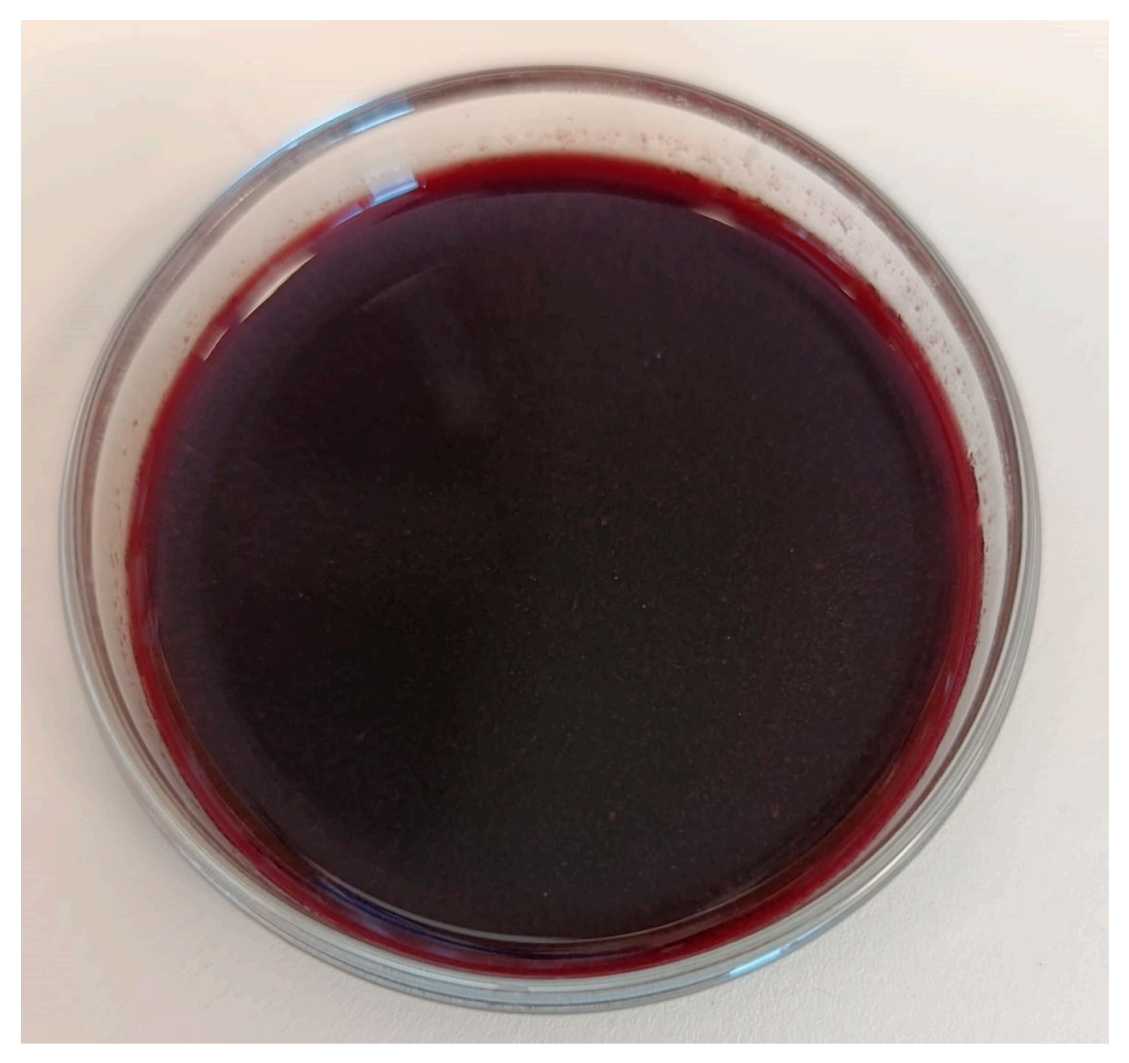
Representative photograph of the elderberry (*Sambucus nigra* L.) pulp used for the preparation of kefir ice cream formulations.

The physicochemical characteristics and antioxidant properties of elderberry pulp are presented in Table 2, while a visual representation of the elderberry pulp is shown in Figure 1. The pulp contained 9.36% total solids, 0.81% ash, and 0.66% protein, indicating its high moisture content, which is consistent with the compositional characteristics generally reported for *Sambucus nigra* fruits [34,35]. The pulp exhibited a pH of 4.31 and a titratable acidity of 0.70% (as citric acid), confirming its naturally acidic nature. Similar pH and acidity values have been reported for elderberry fruits by Lee and Finn [36] and Veberic, et al. [21]. The acidic composition of elderberry is mainly associated with the presence of organic acids, particularly malic and citric acids, which contribute to microbial stability and help preserve anthocyanin pigments during food processing. Therefore, elderberry pulp has the potential to improve both the technological stability and the functional quality of fermented dairy products. Color analysis revealed a low L* value (24.51), together with a positive a* value (1.63) and a nearly neutral b* value (0.04), indicating the characteristic dark purple appearance of elderberry. This distinctive color is primarily attributed to the high concentration of anthocyanins, especially cyanidin derivatives, which are the predominant pigments in elderberry fruits [37,38]. These pigments not only provide an attractive natural color but also contribute substantially to the antioxidant properties of the fruit. The elderberry pulp showed a total phenolic content of 12.98 mg GAE/g FW, together with DPPH radical scavenging activity of 3.86 mg TE/g FW and a FRAP value of 7.00 µmol TE/g FW, indicating its antioxidant potential. The TPC obtained in the present study was higher than the values of 3.64 and 5.10 mg GAE/g FW reported by Lee and Finn [36] for the Haschberg cultivar in two different growing seasons. Similarly, TPC values ranging from 2.90 to 5.01 mg GAE/g FW have been reported among different elderberry accessions by Ozgen et al. [39]. In the latter study, FRAP and DPPH activities ranged from 13.4 to 31.7 and 5.4 to 16.9 µmol TE/g FW, respectively. The FRAP value obtained in the present study was lower than this reported range, whereas the DPPH value, when expressed on the same molar Trolox basis (approximately 15.4 µmol TE/g FW), was within the reported range. These variations may be associated with differences in species and cultivar, growing conditions and season, fruit maturity, processing, extraction procedures, and analytical conditions. Recent studies further support the variability in the phenolic composition of elderberry fruits. Tsanova-Savova et al. [40] reported a total phenolic content of 16.88 mg GAE/g in methanolic extracts of *S. nigra* fruits, while Januškevičė et al. [41] demonstrated substantial cultivar-dependent variation in phenolic content and antioxidant activity among *S. nigra* fruits, including the Haschberg cultivar. Overall, the present results support the potential of elderberry pulp as a source of bioactive compounds for incorporation into functional dairy products.

### 3.2. Physicochemical Properties of Kefir Ice Cream Mixes

Table 3 presents the physicochemical properties of kefir ice cream mixes formulated with different concentrations of elderberry pulp. The physicochemical properties of the commercial kefir used in the preparation of the ice cream mixes are also provided as reference information. The incorporation of kefir and elderberry pulp significantly affected the pH, titratable acidity, and fat content of the mixes (*p* < 0.05), whereas no significant differences were observed in protein, total solids, or ash contents (*p* > 0.05).

The CO sample exhibited the highest pH (6.55) and the lowest titratable acidity (0.16%), while the addition of kefir markedly reduced the pH to 5.54 in the KF sample. Increasing the elderberry pulp concentration further decreased the pH, reaching 5.16 in EP9, whereas titratable acidity increased to 0.41%. These changes can be attributed to the combined effects of the organic acids naturally present in elderberry and the acidic metabolites produced during kefir fermentation. Although the commercial kefir used in the formulation (Table 3) exhibited a pH of 4.50 and a titratable acidity of 0.85%, the final ice cream mixes showed higher pH values because milk, cream, and other ingredients exerted a buffering effect, partially neutralizing the acidity of the fermented ingredient. Similar reductions in pH and increases in titratable acidity have been reported in fermented dairy products enriched with phenolic-rich fruit ingredients [5,19].

Fat content was significantly higher in the CO sample (4.65%) than in the kefir-containing formulations (3.60–3.90%). This reduction is primarily associated with the lower fat content of both kefir and elderberry pulp compared with cream, resulting in a slight dilution of the fat fraction in the final formulation. Comparable decreases in fat percentage following the incorporation of fruit-derived ingredients have also been reported in functional ice cream formulations [42,43].

In contrast, protein, total solids, and ash contents were not significantly affected by elderberry pulp addition. Protein content ranged from 2.75 to 2.93%, total solids from 31.73 to 32.12%, and ash from 0.73 to 0.85%, indicating that the replacement level of elderberry pulp was insufficient to alter the overall composition of the mixes. Maintaining a relatively constant total solids content is technologically important because the balance between water, fat, proteins, sugars, and other solids largely determines the structural stability and processing performance of ice cream systems [44]. Therefore, the observed differences in the subsequent rheological, melting, and textural properties are more likely to be associated with compositional interactions introduced by kefir and elderberry bioactive compounds than with changes in the overall solids content.

### 3.3. Color Properties

**Figure 2:**
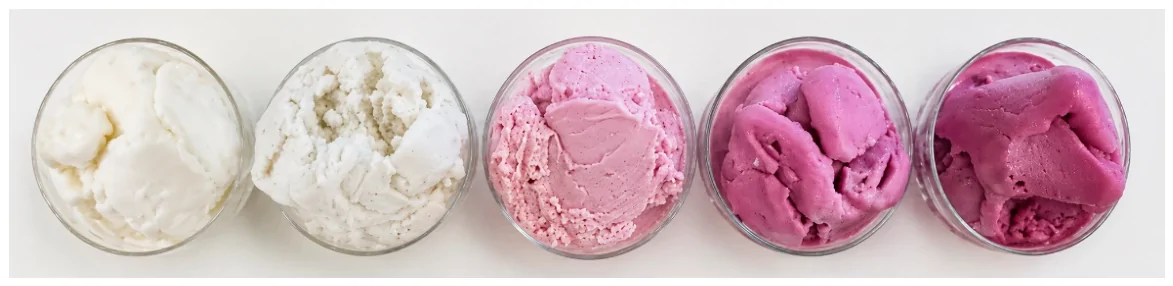
Representative photographs of kefir ice cream formulations. From left to right: control ice cream without kefir and elderberry pulp (CO), kefir control (KF), and kefir ice creams containing 3% (EP3), 6% (EP6), and 9% (EP9) elderberry pulp.

The color characteristics of kefir ice creams are presented in Table 4, while representative photographs of the formulations are shown in Figure 2. The incorporation of elderberry pulp significantly affected all color parameters (*p* < 0.05), indicating that the fruit markedly altered the visual appearance of the products.

A progressive decrease in L* values was observed with increasing elderberry pulp concentration. While the CO sample exhibited the highest lightness (90.11), the value decreased to 46.93 in EP9, producing a noticeably darker product. This reduction is primarily attributed to the high concentration of anthocyanins naturally present in elderberry, which impart an intense purple-red color and reduce the overall lightness of the ice cream matrix [15,37]. The lower L* value of the KF sample compared with CO also indicates that kefir itself slightly influenced product color before elderberry incorporation. The a* values increased significantly from negative values in the control samples to 26.79 in EP9, demonstrating a concentration-dependent enhancement of redness. Anthocyanins, particularly cyanidin-based pigments, are the dominant phenolic compounds responsible for the characteristic red-purple coloration of elderberry fruits [21,38]. Therefore, increasing elderberry pulp levels resulted in a proportional intensification of the red color, suggesting that the color contribution of these pigments was retained during ice cream manufacture. Nevertheless, anthocyanins are sensitive to processing and storage conditions. Since the elderberry pulp used in the present study was pasteurized before incorporation, some degradation of these pigments may have occurred during heat treatment. Oancea et al. [45] reported the thermal degradation of polyphenolic compounds, including anthocyanins, in elderberry extracts, while Baeza et al. [46] demonstrated that the storage stability of elderberry anthocyanins and color characteristics is strongly influenced by storage conditions. Therefore, further studies evaluating anthocyanin retention during pulp pasteurization, freezing, and subsequent frozen storage would be valuable for determining the long-term color and functional stability of elderberry-enriched kefir ice cream under industrial storage conditions.

The b* values shifted from positive values in the control samples to negative values following elderberry incorporation, indicating a reduction in yellow tones and a greater contribution of blue-purple hues. However, unlike L* and a*, the b* values did not exhibit a strictly concentration-dependent response. EP6 showed a less negative b* value (−0.43) than EP3 (−1.71), while EP9 exhibited an intermediate value (−1.02). This non-monotonic pattern may be related to the complex behavior of elderberry pigments within the dairy matrix, since interactions between anthocyanins and milk proteins can influence anthocyanin color expression and stability [47]. In addition, differences in pigment distribution and sample variability may have contributed to the relatively small fluctuations observed among the elderberry-containing formulations. Notably, the b* value of EP9 was not significantly different from either EP3 or EP6 (*p* > 0.05), indicating partial statistical overlap among these treatments. Therefore, the observed variation in b* should be interpreted cautiously rather than as a direct concentration-dependent response to elderberry pulp addition. Similar changes in L*, a*, and b* values have been reported following the incorporation of anthocyanin-rich fruits such as elderberry, blackberry, and aronia into dairy products [15,48]. More recently Pascariu, et al. [18] reported a concentration-dependent decrease in L* and increase in a* values in yogurt supplemented with elderberry extract, consistent with the darker and more intensely red appearance observed in the present study. Overall, elderberry pulp acted as an effective natural colorant, producing darker and more intensely red-purple kefir ice creams.

### 3.4. Melting Properties

The melting characteristics of kefir ice creams are presented in Table 5. Both melting percentage and first dripping time were significantly affected by the incorporation of kefir and elderberry pulp (*p* < 0.05), indicating that formulation changes altered the structural stability of the frozen matrix.

The CO sample exhibited the longest first dripping time (626.67 s), whereas the incorporation of kefir markedly reduced this value to 328.00 s in the KF sample. Among the elderberry-containing formulations, first dripping time ranged from 274.50 to 401.67 s, with EP3 showing the greatest resistance to initial melting. The shorter dripping times observed in kefir-containing samples may be associated with modifications in the serum phase and fat network resulting from fermentation, which influence heat transfer and the initiation of melting [44,49].

In contrast, the overall melting percentage showed a different trend. The highest melting value was observed in the CO sample (85.35%), whereas EP9 exhibited the lowest melting percentage (74.29%). The lower melting percentage observed in EP9 may be associated with compositional and structural changes introduced by elderberry pulp. Based on the known composition of elderberry and previous studies on fruit-enriched frozen systems, fruit-derived pectic substances and dietary fiber could contribute to water binding and reduced water mobility, thereby potentially affecting melting behavior. Similar improvements in melting stability have been reported following the incorporation of fiber-rich fruit ingredients into ice cream formulations [42,50]. Such effects are generally attributed to the water-binding capacity and structural stabilization provided by hydrocolloids and dietary fibers, which reduce melt-down and improve the stability of the frozen matrix [51].

Interestingly, first dripping time and melting percentage did not exhibit a direct linear relationship. For example, EP3 showed a relatively long first dripping time but a melting percentage comparable to that of the control sample, whereas EP9 presented the lowest melting percentage despite an intermediate first dripping time. This finding indicates that the initiation of melting and the subsequent melting rate are governed by different structural factors. While first dripping time is influenced by the integrity of the surface fat network and the onset of ice crystal melting, the overall melting behaviour depends on multiple factors, including air cell distribution, ice crystal structure, serum phase viscosity, and water-binding capacity [28]. Similar observations have been reported by Terzioğlu and Macit [48] who found that increasing the concentration of plant extract prolonged both the first dripping time (1110–1746 s) and the complete melting time (5170–7401 s), indicating that compositional modifications affecting mix viscosity and structural organization can significantly improve melting resistance. Collectively, these findings suggest that elderberry pulp, particularly at the highest supplementation level, may have contributed to improved melting resistance through changes in water distribution and the structural organization of the frozen matrix. This interpretation is further supported by the SEM observations presented in Section 3.8, which revealed distinct differences in matrix organization among the formulations. Although EP9 exhibited a more open porous network than EP6, the matrix remained continuous and well organized, which may have contributed to its lower melting percentage. Similarly, the relatively homogeneous and interconnected network observed in EP3 may explain its longer first dripping time, indicating that different microstructural arrangements can influence the initiation of melting and the subsequent melting process in different ways. These findings suggest that the melting behavior of kefir ice cream may be influenced by changes in water distribution and by the overall organization and continuity of the frozen matrix following elderberry pulp incorporation.

### 3.5. Physical Properties

The physical properties of kefir ice creams are presented in Table 6. Elderberry pulp significantly influenced overrun and density (*p* < 0.05), whereas its effect on viscosity was less pronounced. The viscosity of the ice cream mixes ranged from 2467.5 to 3015.0 cP. Although the KF sample exhibited the highest viscosity and EP9 the lowest, no concentration-dependent trend was observed with increasing elderberry pulp addition. This suggests that the relatively low incorporation levels (3–9%) of elderberry pulp did not substantially alter the flow behaviour of the ice cream mixes. Considering the substantial increase in viscosity observed in the KF sample, the limited effect of elderberry pulp may indicate that viscosity was influenced by multiple matrix components, including milk proteins, fat globules, stabilizers, and the aqueous phase rather than by the added plant material alone. Similar observations have been reported for fruit-enriched frozen dairy desserts, where viscosity is governed not only by dietary fiber but also by the interactions among milk proteins, fat globules, stabilizers, and the serum phase [6,44]. Likewise, Elkot et al. [52] reported that incorporating heart of date palm into low-fat camel milk ice cream enhanced the rheological properties of the mix through the formation of hydrated biopolymer networks and stronger interactions between milk proteins and plant-derived polysaccharides, although the extent of these effects depended on the level of ingredient incorporation. Similarly, Soukoulis et al. [53] demonstrated that the combined action of soluble solids and insoluble dietary fiber increased the viscosity and consistency of fiber-enriched ice cream mixes, highlighting that the rheological response is determined by the overall matrix composition rather than by fiber concentration alone.

More pronounced differences were observed in overrun. The KF sample exhibited the lowest overrun (28.99%), whereas the highest value was obtained for EP6 (62.36%). These values fall within the wide range reported for kefir ice cream and fruit-enriched frozen dairy products. For example, Al et al. [54] reported overrun values of 10.06–21.05% in kefir ice cream formulated with date puree as a sugar substitute, whereas Januário, et al. [26] obtained considerably higher values (87.5–118.0%) in fruit-flavored kefir ice cream sweetened with honey, demonstrating that formulation composition and processing conditions markedly influence air incorporation. Likewise, Elkot, et al. [52] observed that the incorporation of heart of date palm into low-fat ice cream increased overrun by improving the stability of the ice cream mix. In the present study, the improved air incorporation following elderberry pulp addition may be associated with the presence of pectic substances and soluble polysaccharides, which increase the stability of the continuous phase and facilitate air-cell retention during freezing. Similarly, Al, et al. [54] emphasized that overrun is strongly influenced by mix viscosity and pH, and that an optimum viscosity is required for efficient air incorporation, whereas excessively low or high viscosity impairs the formation and stabilization of air cells. Adequate air incorporation is essential for producing a lighter texture and improving the sensory quality of ice cream [4,44].

Density values showed an inverse trend to overrun, decreasing from 0.72 g/cm^3^ in KF to 0.55 g/cm^3^ in EP6. The density values obtained in the present study were within the range reported for functional ice creams enriched with plant-derived ingredients. For example, Khalil, et al. [29] reported specific gravity values of 0.72–0.73 g/cm^3^ in beetroot-enriched ice cream, whereas Goraya and Bajwa [55] observed values ranging from 0.532 to 0.645 g/cm^3^ in ice cream fortified with different forms of Indian gooseberry (*Phyllanthus emblica*), including shreds, pulp, preserve, candy, and powder. The lower density observed in the elderberry pulp-containing samples can be attributed to greater air incorporation and retention within the frozen matrix, as reflected by the corresponding increase in overrun. This inverse relationship between density and overrun has been widely recognized in ice cream systems, where increased air incorporation reduces the mass per unit volume and produces a lighter frozen structure [44]. Consequently, elderberry pulp primarily modified the physical structure of kefir ice cream by improving aeration efficiency rather than by markedly altering mix viscosity. When considered together with the melting results, these findings indicate that viscosity, overrun, density, and melting behavior were interrelated but did not follow a simple linear relationship. Mix viscosity can influence air incorporation and air-cell stabilization during freezing, whereas increased overrun generally reduces product density and modifies the structural organization of the frozen matrix. In turn, the amount and distribution of incorporated air, together with serum-phase viscosity and water binding, can affect heat transfer and melt-down behavior. In the present study, EP6 exhibited the highest overrun and lowest density, whereas EP9 showed the lowest melting percentage despite having a lower viscosity and intermediate overrun. This pattern suggests that melting stability was determined by the combined effects of aeration, continuous-phase properties, water immobilization, and matrix organization rather than by any single physical parameter.

### 3.6. Textural Properties

The textural properties of kefir ice creams are presented in Table 7. The incorporation of kefir and elderberry pulp significantly affected both hardness and stickiness (*p* < 0.05).

The KF sample exhibited the highest hardness (2024 g), representing a marked increase compared with the CO sample (714 g). The higher hardness observed after kefir incorporation may be attributed to acidification-induced rearrangement of the casein network during fermentation together with the formation of a more compact protein matrix, which may have increased the mechanical resistance of the frozen structure. Following the addition of elderberry pulp, hardness decreased to values between 1136.8 and 1662.2 g, although the response was not concentration-dependent. Similar formulation-dependent changes in hardness have been reported for kefir ice cream flavored with mint, where hardness ranged from 69.23 to 106.19 g, confirming that plant-derived ingredients can markedly influence the textural properties of kefir ice cream [25]. Toommuangpak and Thaiudom [56] also reported variations in hardness among yogurt ice cream formulations, supporting the view that hardness is influenced by multiple compositional and structural factors. Although the absolute hardness values in the present study were considerably higher, differences in formulation, freezing conditions, and instrumental measurement parameters among studies may account for these discrepancies.

In the present study, hardness decreased markedly in EP3, increased again in EP6, and declined in EP9, indicating a non-linear textural response to elderberry pulp concentration. This pattern may reflect changes in water distribution and matrix organization associated with the incorporation of elderberry pulp. The dietary fiber and pectic substances naturally present in elderberry may contribute to these changes by influencing water distribution within the unfrozen serum phase and, potentially, the organization of the structural matrix. At the intermediate supplementation level (EP6), these combined effects may have contributed to the greater structural rigidity and the corresponding increase in hardness. However, at the highest supplementation level (EP9), the greater amount of pulp may have disrupted the continuity of the protein–fat network and phase distribution, potentially contributing to a softer structure. Similar non-linear textural responses have been reported in frozen dairy products enriched with fruit-derived fibers and hydrocolloid-rich ingredients [53,57].

Stickiness ranged from −272.9 to −389.6 g. The KF sample showed the highest stickiness, while the incorporation of elderberry pulp significantly reduced stickiness in all fortified formulations. The reduction in stickiness may be related to changes in water distribution and the properties of the unfrozen serum phase following elderberry pulp incorporation. Dietary fiber is known to possess water-retention and structure-building properties, which may modify the physical state and mobility of water within frozen dairy matrices [58]. Such changes may reduce the amount of mobile material available at the product surface and consequently decrease adhesion between the ice cream and the probe during texture analysis.

The combined effects of kefir fermentation and elderberry pulp substantially modified the textural profile of the ice cream. While kefir increased product hardness, elderberry supplementation generally produced a softer and less sticky structure. These changes are likely associated with modifications in water distribution and matrix organization, which play a key role in determining the mechanical properties and eating quality of frozen dairy products [28,59].

### 3.7. Total Phenolic Content and Antioxidant Activity

The total phenolic content (TPC) and ferric reducing antioxidant power (FRAP) of kefir ice creams are presented in Table 8. Elderberry pulp significantly enhanced both parameters (*p* < 0.05), demonstrating its effectiveness as a natural source of antioxidant compounds. The KF sample exhibited the lowest TPC (306.81 mg GAE/100 g DM), whereas TPC increased progressively with increasing elderberry pulp concentration, reaching 694.16 mg GAE/100 g DM in EP9. This concentration-dependent increase is consistent with the high phenolic content of the elderberry pulp used in the present study (12.98 mg GAE/g FW) and confirms that the fruit served as the primary source of phenolic compounds in the ice cream formulations. Elderberry is recognized as a rich source of anthocyanins, phenolic acids, and flavonoids, particularly chlorogenic acid, rutin, and quercetin derivatives, which contribute substantially to its antioxidant properties [15,21]. Comparable concentration-dependent increases in TPC have been reported for functional ice creams enriched with berry extracts. Terzioğlu and Macit [48] observed that the incorporation of *Sambucus nigra* and *Aronia melanocarpa* extracts increased TPC from 54.52 to 749.33 mg GAE/kg, with the highest values obtained at the greatest extract concentration. These findings collectively demonstrate that the enrichment of ice cream with phenolic-rich natural ingredients consistently enhances its phenolic profile, although the magnitude of the increase depends on the type, concentration, and phenolic composition of the added material.

A similar trend was observed for FRAP values. Antioxidant activity increased from 17.54 µmol TE/100 g DM in the KF sample to 49.70 µmol TE/100 g DM in EP9, indicating a marked improvement in the ferric reducing capacity of the products following elderberry supplementation. The close correspondence between TPC and FRAP values suggests that phenolic compounds were the major contributors to the antioxidant activity of the ice cream. A strong positive correlation was observed between total phenolic content and FRAP antioxidant capacity (Pearson’s r = 0.952, *p* < 0.001), indicating that the increase in phenolic compounds was closely associated with enhanced ferric-reducing antioxidant capacity. Similarly, Terzioğlu and Macit [48] reported that increasing the concentration of *Sambucus nigra* and *Aronia melanocarpa* extracts enhanced the antioxidant capacity of functional ice creams, as evidenced by progressively lower IC_50_ values for both DPPH and ABTS radical scavenging assays, together with higher TPC. The enhancement of antioxidant activity is likely associated with the ability of phenolic compounds to donate electrons and reduce ferric ions, thereby increasing the reducing power of the ice cream matrix. In particular, anthocyanins and hydroxycinnamic acids, which are abundant in elderberry, possess strong redox properties and are considered major contributors to FRAP activity [35,38].

### 3.8. Microstructural Properties

**Figure 3:**
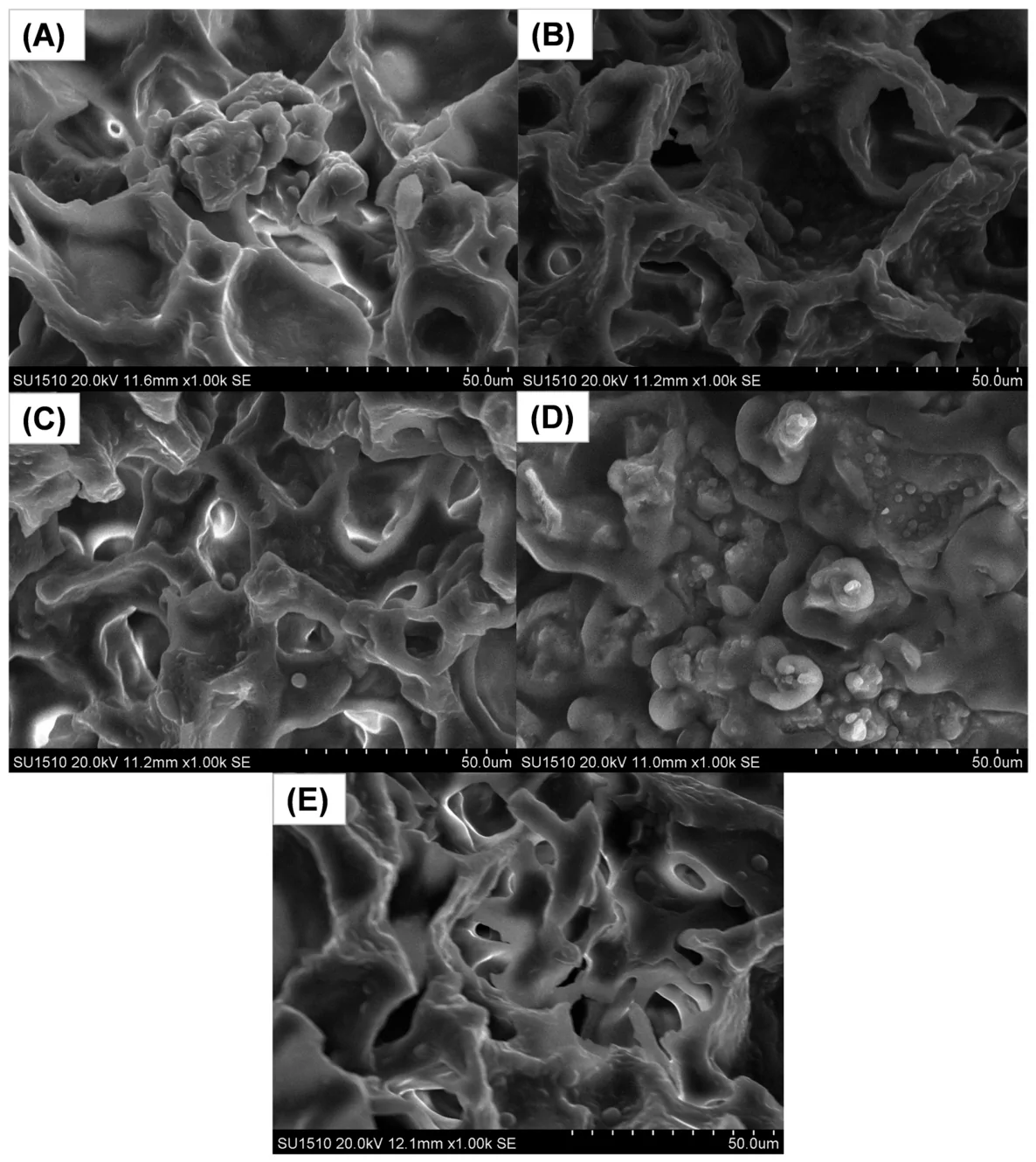
Scanning electron microscopy (SEM) micrographs of kefir ice creams containing different concentrations of elderberry pulp (1000× magnification). The micrographs illustrate formulation-dependent differences in matrix organization, with a relatively denser structure observed in EP6 and more open yet continuous porous structures in EP3 and EP9. (**A**) Control ice cream without kefir and elderberry pulp (CO); (**B**) kefir control ice cream (KF); (**C**) kefir ice cream containing 3% elderberry pulp (EP3); (**D**) kefir ice cream containing 6% elderberry pulp (EP6); and (**E**) kefir ice cream containing 9% elderberry pulp (EP9). Micrographs were obtained at an accelerating voltage of 20 kV and a magnification of 1000×. Scale bar = 50 μm.

Scanning electron microscopy (SEM) images of kefir ice creams containing different concentrations of elderberry pulp are presented in Figure 3. All formulations exhibited a porous three-dimensional network consisting of interconnected protein aggregates surrounding voids generated during freeze-drying by the sublimation of ice crystals. Such porous architectures are characteristic of frozen dairy systems and reflect the spatial organization of milk proteins, partially coalesced fat globules, air cells, and the unfrozen serum phase [44,60]. The CO sample exhibited a relatively compact and continuous matrix with smaller and more uniformly distributed pores, whereas the KF sample showed a more open and heterogeneous network, indicating that kefir fermentation altered the organization of the protein matrix through acidification-induced rearrangement of casein micelles. Similar structural modifications have been reported to influence the mechanical properties and stability of fermented dairy products by altering protein connectivity and serum-phase organization [61].

Although elderberry pulp did not fundamentally alter the overall porous architecture, clear differences in matrix organization were observed among the supplemented samples. EP3 displayed a relatively homogeneous and interconnected network, suggesting uniform dispersion of the fruit components throughout the matrix. In contrast, EP6 exhibited localized dense regions and particulate aggregates, producing a more compact microstructure that may reflect interactions between fruit-derived structural components and the dairy matrix, potentially contributing to localized water immobilization [62]. The EP9 sample exhibited a more open yet continuous porous network, which may suggest that increasing the elderberry concentration beyond an intermediate level promoted a more homogeneous structural arrangement rather than further compaction. This observation was consistent with the physical measurements, where EP9 exhibited lower hardness than EP6 despite its higher pulp content. The SEM observations closely agreed with the physical and textural characteristics of the ice creams. The denser matrix observed in EP6 corresponded to its relatively higher hardness, whereas the more open and homogeneous structures of EP3 and EP9 were associated with lower hardness, reduced density, and higher overrun. These microstructural differences were also consistent with the melting properties discussed in Section 3.4. In particular, the continuous and well-organized porous network observed in EP9 may have contributed to its lower melting percentage, whereas the relatively homogeneous interconnected structure of EP3 may have contributed to its longer first dripping time. These findings indicate that the melting behavior of kefir ice cream is influenced not only by the composition of the mix but also by the spatial organization and continuity of the frozen matrix. Taken together, the distinctive behavior of EP6 across several physical and structural parameters suggests a non-linear response to elderberry pulp concentration. At this intermediate incorporation level, pulp–matrix interactions may have favored localized structural reinforcement, whereas further increasing the pulp level to 9% appeared to reorganize rather than further compact the matrix. Therefore, the behavior of EP6 is more appropriately interpreted as a formulation-dependent structural response rather than a simple concentration-dependent effect, although experimental variability may also have contributed to some of the observed differences. Similar relationships between microstructure, melting characteristics, and structural stability have been reported in previous studies on frozen dairy systems [60,61,63]. Overall, the SEM results suggest that elderberry pulp was associated with formulation-dependent modifications in matrix organization without compromising the overall integrity of the frozen dairy system.

### 3.9. Thermal Properties

Thermal properties of kefir ice creams containing different concentrations of elderberry pulp are presented in Table 9. All formulations exhibited a characteristic endothermic transition corresponding to the melting of ice crystals. The onset, peak, endset temperatures, and melting enthalpy (ΔH) varied among the formulations, indicating that the incorporation of kefir and elderberry pulp influenced the thermal behaviour of the frozen matrix. The onset temperature ranged from −32.10 to −20.56 °C, with the lowest value observed for the KF sample and the highest for EP3. Peak temperatures ranged from −21.53 to −10.75 °C, whereas endset temperatures ranged from −15.42 to −8.03 °C. These thermal transitions reflect differences in the melting behaviour of ice crystals and indicate that the composition of the ice cream matrix influenced the stability and melting characteristics of the frozen water fraction. The presence of proteins, sugars, and other soluble constituents alters the freezing profile by modifying the distribution of water between frozen and unfrozen phases [64]. The melting enthalpy (ΔH) values ranged from 158.40 to 208.40 J/g. The KF sample exhibited the highest ΔH value (208.40 J/g), whereas the elderberry-containing formulations showed values between 158.40 and 161.20 J/g. Since melting enthalpy is primarily related to the amount of freezable water present in a frozen system, the lower ΔH values of the elderberry-containing formulations suggest that a smaller fraction of water was present as ice and therefore required less energy for melting. This effect may be associated with the water-binding capacity of fruit-derived polysaccharides and other soluble constituents present in elderberry pulp. These components may interact with water and reduce its availability for ice formation, potentially increasing the proportion of unfrozen or less readily freezable water within the matrix. In addition, the soluble solids contributed by elderberry pulp may increase the concentration of dissolved substances in the aqueous phase, which can depress the freezing point and further modify the balance between freezable and unfrozen water. Consequently, the lower ΔH values observed after elderberry incorporation may reflect a reduction in the amount of ice formed during freezing and a corresponding increase in water associated with the non-frozen phase. Similar effects have been reported in frozen dairy products enriched with plant-derived ingredients containing soluble solids and hydrocolloid-like components, where increased water binding and changes in water mobility influenced ice crystallization and thermal transitions [62,65]. The DSC observations were generally consistent with the physical and microstructural characteristics of the ice creams. The elderberry-containing formulations exhibited lower ΔH values together with differences in overrun, density, and matrix organization observed by SEM. Collectively, these findings suggest that elderberry pulp may have influenced the distribution of water within the frozen matrix, thereby contributing to variations in thermal behaviour. Overall, these findings are consistent with the established importance of freezable and non-freezable water distribution in determining the thermal properties of frozen food systems [64].

### 3.10. Volatile Aroma Compounds

A total of 25 volatile compounds were tentatively identified based on mass spectral library matching in the kefir ice cream formulations, including aldehydes, ketones, alcohols, esters, organic acids, and terpenes (Table 10). Ketones represented the largest volatile group in all formulations. However, the relative contribution of the remaining chemical groups differed among the samples. Aldehydes constituted the second largest group in CO, esters and organic acids showed comparable contributions in KF, and organic acids became more prominent in the elderberry-containing formulations. These differences suggest that the observed volatile profiles may reflect the combined influence of the dairy matrix, kefir fermentation, and elderberry pulp incorporation. Among the ketones, 2-heptanone showed the highest relative peak area, ranging from 19.05 to 37.69%. Methyl ketones are characteristic aroma compounds of fermented dairy products and may be generated through fatty acid β-oxidation and microbial catabolism. They have been associated with buttery, creamy, fruity, and blue cheese-like aroma notes to fermented milk products [66,67]. In the present study, the mean relative abundance of 2-heptanone was 37.69% in CO, 19.85% in KF, and 19.05–19.54% in the elderberry-containing formulations. Meral-Aktaş et al. [68] also reported 2-heptanone among the characteristic volatile compounds of kefir produced from different animal milks, supporting the relevance of methyl ketones to the aroma of fermented dairy products. Acetoin displayed a different distribution. Its relative peak area was 1.00% in CO and ranged from 16.25 to 20.58% in the kefir-containing formulations. Acetoin can be produced through citrate and pyruvate metabolism by lactic acid bacteria, particularly species of *Lactococcus* and *Leuconostoc*, and has been associated with buttery and creamy flavor notes [66]. Lu et al. [69] similarly reported acetoin as an important odor-active compound in kefir, together with butanoic acid, hexanal, and 2,3-butanedione.

Among the aldehydes, acetaldehyde showed the highest relative peak area, ranging from 3.46 to 6.02%. Acetaldehyde has been associated with the fresh, green, and yogurt-like aroma of fermented dairy products, whereas hexanal and nonanal are commonly linked to lipid oxidation and fatty acid degradation [67]. The occurrence of these tentatively assigned compounds across the formulations is consistent with volatile compounds previously associated with dairy matrices and fermented dairy products. Similar aldehyde profiles have been reported in kefir samples analysed by HS-SPME-GC-MS [68].

The organic acid fraction differed notably among the formulations. Acetic acid represented 1.00% of the relative peak area in CO, 6.31% in KF, and 12.71–17.32% in the elderberry-containing formulations. Butyric, hexanoic, octanoic, and decanoic acids were also tentatively identified in varying proportions. Because elderberry pulp was incorporated after kefir fermentation, the differences observed among EP3, EP6, and EP9 are more appropriately attributed to the direct contribution of fruit-derived volatile constituents and to compositional changes caused by blending the elderberry matrix with kefir ice cream, rather than to altered microbial fermentation. Fruit addition has similarly been reported to modify the volatile acid composition of fermented dairy products [70,71].

Among the esters, ethyl pyruvate was detected in all formulations, with relative peak areas ranging from 3.76 to 8.18%, and could potentially be associated with sweet and fruity aroma notes. Notably, heptanal dimethyl acetal, hydroxyacetone, and isopropyl acetate were not detected in the EP9 formulation. However, their absence cannot be attributed solely to the higher elderberry pulp concentration. Heptanal dimethyl acetal generally decreased with elderberry pulp incorporation and was no longer detected in EP9, suggesting that increasing replacement of the dairy matrix with elderberry pulp may have contributed to its reduced relative abundance. In contrast, hydroxyacetone was inconsistently detected across the formulations, being absent in KF and EP6 as well as EP9, while isopropyl acetate did not show a concentration-dependent trend, reaching its highest relative abundance in EP6 before becoming undetectable in EP9. Therefore, these changes are more likely to reflect compound-specific responses to alterations in matrix composition rather than a uniform concentration-dependent effect of elderberry pulp. Interactions of volatile compounds with proteins, lipids, carbohydrates, and polyphenols may influence their retention and release from complex food matrices, potentially affecting their abundance in the headspace during HS-SPME-GC/MS analysis [72]. Accordingly, the non-detection of these compounds in EP9 may indicate that their concentrations in the analyzed headspace were below the reporting threshold under the present analytical conditions, rather than their complete absence from the product.

Limonene was the only terpene tentatively identified, with relative peak areas ranging from 0.50 to 1.61%. Because limonene was also detected in the control formulations, its presence cannot be attributed exclusively to elderberry pulp. Nevertheless, compounds tentatively assigned as terpenes and esters may be associated with volatiles originating from different components of the formulation and may contribute to aroma complexity; however, their actual sensory contribution cannot be established from the present GC-MS data alone [70].

Overall, ketones dominated the volatile composition of all formulations, while the relative proportions of aldehydes, alcohols, esters, organic acids, and terpenes varied among samples. The kefir-containing formulations showed higher mean relative proportions of acetoin and acetic acid than CO, while elderberry pulp incorporation was associated with further differences in organic acids and other tentatively identified volatile compounds. These patterns suggest that both kefir fermentation and post-fermentation elderberry pulp incorporation may have influenced the final volatile profile. This interpretation is consistent with previous studies showing that starter cultures shape the basic volatile profile of fermented dairy products, while plant-derived ingredients further modify the abundance and diversity of aroma compounds [73]. Nevertheless, because compound assignments were based on mass spectral library matching without confirmation by retention indices or authentic standards, interpretations regarding individual compounds and their sensory contributions should be considered tentative.

**Table 10:** Tentatively identified volatile compounds and their relative peak areas (%) in kefir ice creams containing different concentrations of elderberry pulp.

Compound	RT (min)	Reported Aroma Descriptor	CO	KF	EP3	EP6	EP9
Aldehydes							
Acetaldehyde	1.47	Pungent, ethereal	6.02 ± 2.00	4.03 ± 0.30	4.50 ± 0.65	3.46 ± 0.88	3.57 ± 0.40
Heptanal dimethyl acetal	2.01	Green, herbaceous, nutty	1.78 ± 0.00	0.93 ± 0.10	0.46 ± 0.17	0.55 ± 0.00	-
2-Methylpropanal	2.02	Dark chocolate	0.84 ± 0.00	0.66 ± 0.15	0.78 ± 0.20	0.81 ± 0.00	0.41 ± 0.02
Hexanal	5.43	Green, earthy	2.19 ± 0.69	1.89 ± 0.81	1.15 ± 0.01	1.54 ± 0.45	1.54 ± 0.52
Nonanal	16.16	Waxy-green, floral	1.76 ± 1.40	1.28 ± 1.13	0.75 ± 0.13	0.68 ± 0.36	0.61 ± 0.22
Ketones							
Acetone	1.74	Pungent, sweetish, solvent-like	8.15 ± 4.07	6.06 ± 1.95	6.84 ± 0.68	4.92 ± 3.04	4.21 ± 2.60
Hydroxyacetone	2.20	Pungent, sweet-caramelly, ethereal	2.83 ± 0.00	-	2.14 ± 0.00	-	-
2-Pentanone	3.14	Ethereal, fruity	6.49 ± 1.25	3.45 ± 0.86	3.01 ± 0.42	3.25 ± 0.43	2.81 ± 0.43
Acetoin	3.59	Buttery	1.00 ± 0.29	19.52 ± 3.80	20.58 ± 1.35	19.19 ± 2.33	16.25 ± 3.02
2-Heptanone	8.24	Fruity, spicy	37.69 ± 5.47	19.85 ± 3.15	19.54 ± 1.08	19.05 ± 0.69	19.18 ± 0.68
2-Octanone	15.71	Apple-like	7.71 ± 1.08	4.02 ± 1.03	4.37 ± 1.10	4.25 ± 0.77	4.55 ± 0.90
2-Undecanone	22.98	Citrus, fatty, rue-like	1.49 ± 0.35	0.72 ± 0.24	0.83 ± 0.30	0.86 ± 0.30	0.86 ± 0.24
Alcohols							
2-Hexanol	1.64	Characteristic odor	0.54 ± 0.00	0.51 ± 0.00	3.58 ± 0.63	3.81 ± 0.00	1.18 ± 0.00
2-Butanol	1.64	Sweet, fruity	2.11 ± 1.76	2.23 ± 1.21	3.60 ± 0.00	2.08 ± 0.88	2.13 ± 1.56
Isoamyl alcohol	3.94	Alcoholic, fruity, malty	1.07 ± 0.81	0.49 ± 0.10	0.42 ± 0.13	0.32 ± 0.24	0.27 ± 0.10
Butadienol <2,3->	5.36	—	-	0.33 ± 0.00	0.64 ± 0.37	0.58 ± 0.17	2.58 ± 2.95
2-Heptanol	8.60	Fatty, oily	-	1.93 ± 0.00	1.48 ± 0.98	1.01 ± 0.58	1.09 ± 0.77
Esters							
Ethyl pyruvate	2.17	Sweet, floral-fruity, warm	3.76 ± 2.26	8.18 ± 0.28	5.47 ± 0.91	3.84 ± 2.50	7.47 ± 1.09
Isopropyl acetate	2.33	Fruity	1.23 ± 0.00	1.38 ± 0.00	0.91 ± 0.00	2.33 ± 0.00	-
Acids							
Acetic acid	2.32	Vinegar-like	1.00 ± 0.00	6.31 ± 1.37	12.71 ± 2.86	17.32 ± 5.71	16.93 ± 1.20
Butyric acid	5.06	Chessy, rancid	-	0.40 ± 0.00	0.82 ± 0.07	1.47 ± 0.30	1.51 ± 0.08
Hexanoic acid	11.58	Cheesy, sweat-like	-	0.90 ± 0.12	2.01 ± 0.79	2.20 ± 1.24	1.95 ± 0.76
Octanoic acid	18.69	Fatty, unpleasant	0.89 ± 0.40	1.11 ± 0.12	1.79 ± 0.00	1.43 ± 0.42	1.64 ± 0.32
Decanoic acid	25.36	Fatty, rancid	0.63 ± 0.10	0.70 ± 0.24	0.53 ± 0.08	0.57 ± 0.02	0.57 ± 0.06
Terpenes							
Limonene	13.31	Fresh, sweet, citrus-like	1.61 ± 0.41	0.77 ± 0.12	0.50 ± 0.05	0.63 ± 0.08	0.53 ± 0.14

CO: control ice cream (without kefir and elderberry pulp); KF: kefir control ice cream; EP3: kefir ice cream containing 3% elderberry pulp; EP6: kefir ice cream containing 6% elderberry pulp; EP9: kefir ice cream containing 9% elderberry pulp. Values are expressed as mean ± standard deviation (*n* = 3). Only volatile compounds with an average relative peak area ≥ 0.5% are presented. “-” indicates that the compound was not detected or had an average relative peak area below 0.5%. RT: retention time. Reported aroma descriptors were compiled from published literature [74,75,76,77]. These descriptors represent odor characteristics reported for individual volatile compounds and do not necessarily indicate their actual sensory contribution within the kefir ice cream matrix. Volatile compounds were tentatively identified based on mass spectral library matching (FFNSC and NIST 11) without confirmation by retention indices or authentic reference standards.

### 3.11. Sensory Properties

**Figure 4:**
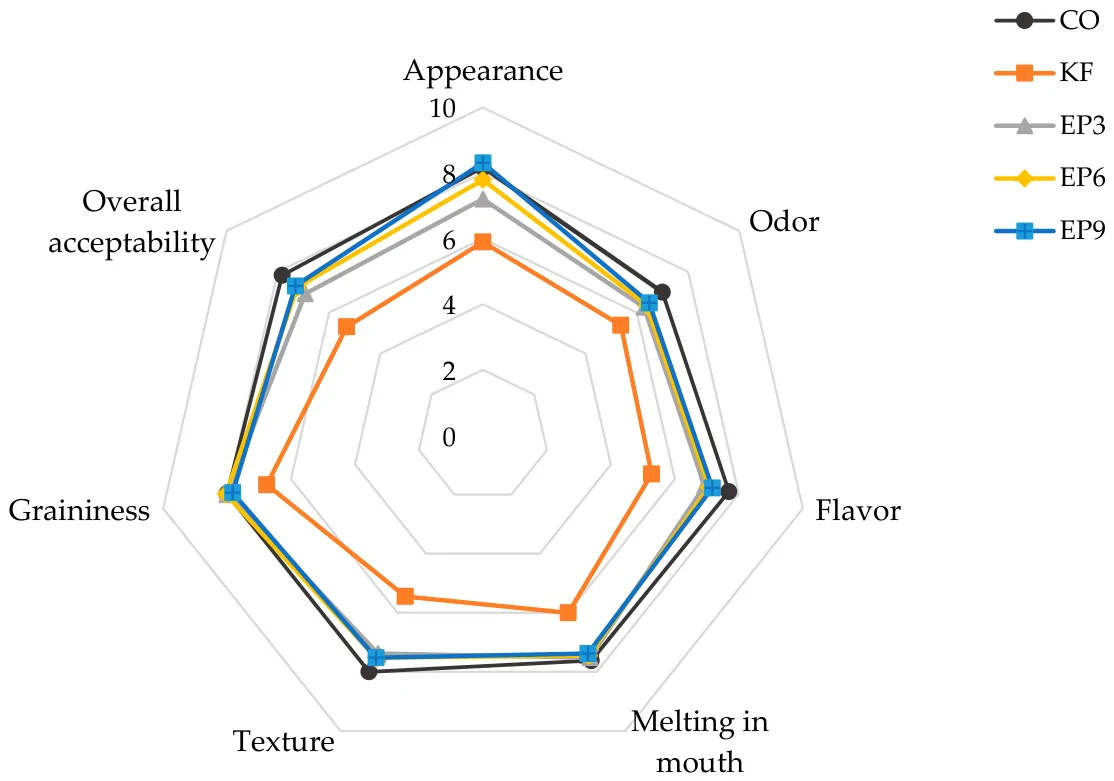
Radar chart showing the sensory profiles of kefir ice cream formulations with different levels of elderberry pulp. Elderberry supplementation improved the sensory profile compared with the kefir control, while EP6 and EP9 showed favorable overall acceptability. Values represent mean sensory scores (*n* = 30) on a 9-point hedonic scale. Standard deviations and statistical comparisons are presented in Table 11.

The sensory evaluation results of kefir ice creams containing different concentrations of elderberry pulp are presented in Table 11. The sensory profiles of the formulations are additionally illustrated using a radar chart in Figure 4 to facilitate visual comparison among the samples. Significant differences were observed among the formulations for all evaluated sensory attributes (*p* < 0.05), demonstrating that kefir fermentation and elderberry supplementation influenced consumer perception. Overall, the CO sample received the highest sensory scores, whereas the KF sample exhibited the lowest ratings for appearance, odor, flavor, texture, and overall acceptability. The incorporation of elderberry pulp significantly improved the sensory quality of kefir ice cream compared with the KF sample. However, the numerical differences among EP3, EP6, and EP9 were generally small, indicating that statistical differences did not necessarily correspond to substantial differences in perceived sensory quality. Thus, the practical sensory effect of increasing elderberry pulp beyond 3% appears limited for most attributes. EP6 and EP9 nevertheless achieved slightly higher overall acceptability scores than EP3 and were statistically comparable to the CO formulation.

Appearance scores were significantly higher in the elderberry-enriched formulations than in the KF sample (*p* < 0.05). Although EP9 showed the highest numerical appearance score, it did not differ significantly from the CO and EP6 formulations. The improved visual perception can be attributed to the attractive purple-red color imparted by anthocyanins, consistent with the instrumental color measurements showing increased redness and reduced lightness. Similarly, elderberry supplementation significantly enhanced odor and flavor compared with the KF sample. These differences may partly reflect changes in the overall aroma profile associated with elderberry incorporation; however, the actual sensory contributions of the individual volatile compounds tentatively identified by HS-SPME-GC/MS cannot be established from the present data.

Texture and melting in mouth also improved following elderberry addition. Compared with the KF sample, all elderberry-enriched formulations exhibited lower hardness together with higher overrun, lower density, and a more homogeneous microstructure, resulting in a smoother and creamier perception. Graininess scores remained relatively high across the formulations, suggesting that elderberry pulp incorporation did not result in a pronounced perception of coarse texture. These findings agree with previous studies reporting improved sensory quality in fruit-enriched kefir and ice cream products [19,59,78]. Overall, the sensory findings were broadly consistent with the instrumental observations, suggesting that elderberry pulp improved the sensory acceptance of kefir ice cream relative to the kefir control. From a practical sensory perspective, increasing the pulp concentration above 3% provided only modest additional improvements, although EP6 and EP9 showed a favorable overall sensory profile.

## 4. Conclusions

This study demonstrated the potential of elderberry (*Sambucus nigra* L.) pulp as a natural ingredient for improving the technological and functional characteristics of kefir ice cream. The incorporation of elderberry pulp enhanced antioxidant capacity and was associated with changes in color, overrun, density, melting behavior, thermal properties, microstructure, volatile aroma profile, and sensory quality without adversely affecting the overall integrity of the frozen dairy matrix. The complementary results obtained from DSC and SEM analyses suggested that elderberry pulp modified water distribution and matrix organization, while HS-SPME-GC/MS analysis indicated differences in the relative distribution of tentatively identified volatile compounds among the formulations. Considering the physicochemical, antioxidant, thermal, microstructural, volatile, and sensory characteristics together, formulations containing 6% and 9% elderberry pulp provided a favorable balance between technological quality and sensory acceptability. From an industrial perspective, these findings suggest that elderberry pulp can be incorporated at 6–9% as a practical formulation range for developing kefir-based frozen desserts with enhanced antioxidant properties and natural red-purple coloration while maintaining acceptable technological and sensory characteristics. Its use may therefore offer manufacturers an opportunity to diversify fermented frozen dairy products through the relatively straightforward incorporation of a fruit-derived functional ingredient into the product formulation. Several limitations should be considered when interpreting these findings. The study was limited to a single elderberry cultivar and selected pulp concentrations, volatile compounds were tentatively identified without confirmation by retention indices or authentic standards, and sensory evaluation was conducted using a semi-trained panel rather than a larger consumer population. Future studies should investigate frozen storage stability, particularly potential changes in elderberry-derived anthocyanins and antioxidant activity, as well as texture and sensory quality over time. The bioaccessibility of phenolic compounds following simulated gastrointestinal digestion, confirmation of key volatile compounds using complementary analytical approaches, and sensory validation in larger consumer populations also warrant further investigation. Further characterization of protein–polyphenol interactions and microstructural organization may also provide deeper insight into the non-linear structural responses observed at different elderberry pulp incorporation levels. Although concentrations above 9% were not selected for detailed evaluation because of adverse effects observed during preliminary formulation trials, alternative formulation strategies may enable higher supplementation levels to be investigated.

## Figures and Tables

**Table 1:** Formulation of kefir ice cream mixes containing different concentrations of elderberry pulp (g per 1000 g mix).

Ingredient (g)	CO	KF	EP3	EP6	EP9
Kefir	0	340	340	340	340
Cow milk	730	390	360	330	300
Cream	80	80	80	80	80
Sucrose	150	150	150	150	150
Skim milk powder	32	32	32	32	32
Salep	4	4	4	4	4
Emulsifier	4	4	4	4	4
Elderberry pulp	0	0	30	60	90
Total	1000	1000	1000	1000	1000

CO: control ice cream (without kefir and elderberry pulp); KF: kefir control ice cream; EP3: kefir ice cream containing 3% elderberry pulp; EP6: kefir ice cream containing 6% elderberry pulp; EP9: kefir ice cream containing 9% elderberry pulp.

**Table 2:** Physicochemical properties of elderberry pulp.

Parameter	Mean ± SD
Total solids (%)	9.36 ± 0.46
Ash (%)	0.81 ± 0.10
Protein (%)	0.66 ± 0.09
pH	4.31 ± 0.01
Titratable acidity (%)	0.70 ± 0.01
L*	24.51 ± 2.26
a*	1.63 ± 0.13
b*	0.04 ± 0.20
TPC (mg GAE/g FW)	12.98 ± 1.23
FRAP (µmol TE/g FW)	7.00 ± 0.08
DPPH (mg TE/g FW)	3.86 ± 0.04

Values are expressed as mean ± standard deviation (*n* = 2). FW: fresh weight. Titratable acidity is expressed as citric acid (%).

**Table 3:** Physicochemical properties of kefir ice cream mixes containing different concentrations of elderberry pulp.

Sample	pH	TA (%)	Fat (%)	Protein (%)	TS (%)	Ash (%)
CO	6.55 ± 0.01 a	0.16 ± 0.05 b	4.65 ± 0.21 a	2.92 ± 0.10 a	31.88 ± 0.06 a	0.73 ± 0.02 a
KF	5.54 ± 0.01 b	0.33 ± 0.00 a	3.75 ± 0.21 b	2.93 ± 0.05 a	31.88 ± 0.11 a	0.85 ± 0.13 a
EP3	5.43 ± 0.01 c	0.36 ± 0.00 a	3.60 ± 0.00 b	2.93 ± 0.05 a	31.91 ± 0.20 a	0.73 ± 0.03 a
EP6	5.26 ± 0.00 d	0.35 ± 0.05 a	3.90 ± 0.00 b	2.86 ± 0.15 a	31.73 ± 0.10 a	0.74 ± 0.01 a
EP9	5.16 ± 0.01 e	0.41 ± 0.00 a	3.60 ± 0.42 b	2.75 ± 0.08 a	32.12 ± 0.77 a	0.75 ± 0.02 a
Kefir	4.50 ± 0.01	0.85 ± 0.03	3.10 ± 0.10	3.20 ± 0.10	12.11 ± 0.68	0.73 ± 0.00

CO: control ice cream mix (without kefir and elderberry pulp); KF: kefir control ice cream mix; EP3: kefir ice cream mix containing 3% elderberry pulp; EP6: kefir ice cream mix containing 6% elderberry pulp; EP9: kefir ice cream mix containing 9% elderberry pulp. Values are expressed as mean ± standard deviation (*n* = 3). Different lowercase letters within the same column indicate significant differences among samples (*p* < 0.05). TA, titratable acidity (expressed as % lactic acid); TS, total solids. The kefir represents the physicochemical characteristics of the kefir used in the preparation of the ice cream mixes.

**Table 4:** Color properties (L*, a*, and b*) of kefir ice creams containing different concentrations of elderberry pulp.

Sample	L*	a*	b*
CO	90.11 ± 1.29 a	−0.43 ± 0.43 d	10.29 ± 0.46 a
KF	82.81 ± 3.62 b	−0.54 ± 0.34 d	8.67 ± 0.98 b
EP3	68.55 ± 0.88 c	12.72 ± 1.22 c	−1.71 ± 0.44 d
EP6	57.97 ± 1.09 d	17.57 ± 0.38 b	−0.43 ± 0.80 c
EP9	46.93 ± 1.92 e	26.79 ± 3.38 a	−1.02 ± 0.89 cd

CO: control ice cream (without kefir and elderberry pulp); KF: kefir control ice cream; EP3: kefir ice cream containing 3% elderberry pulp; EP6: kefir ice cream containing 6% elderberry pulp; EP9: kefir ice cream containing 9% elderberry pulp. Values are expressed as mean ± standard deviation (*n* = 5). Different lowercase letters within the same column indicate significant differences among samples (*p* < 0.05). L* indicates lightness (0 = black, 100 = white), a* redness (+) to greenness (−), and b* yellowness (+) to blueness (−).

**Table 5:** Melting properties of kefir ice creams containing different concentrations of elderberry pulp.

Sample	Melting (%)	First Dripping Time (s)
CO	85.35 ± 3.88 a	626.67 ± 48.42 a
KF	76.98 ± 0.75 b	328.00 ± 96.95 d
EP3	81.29 ± 18.93 a	401.67 ± 207.93 b
EP6	84.02 ± 16.30 a	274.50 ± 16.26 e
EP9	74.29 ± 10.94 b	369.67 ± 43.41 c

CO: control ice cream (without kefir and elderberry pulp); KF: kefir control ice cream; EP3: kefir ice cream containing 3% elderberry pulp; EP6: kefir ice cream containing 6% elderberry pulp; EP9: kefir ice cream containing 9% elderberry pulp. Values are expressed as mean ± standard deviation (*n* = 3). Different lowercase letters within the same column indicate significant differences (*p* < 0.05). Melting (%) represents the proportion of melted ice cream relative to the initial sample weight after the predetermined melting period. First dripping time refers to the time required for the first drop of melted ice cream to fall from the sample.

**Table 6:** Physical and rheological properties of kefir ice creams containing different concentrations of elderberry pulp.

Sample	Viscosity (cP)	Overrun (%)	Density (g/cm^3^)
CO	2642.5 ± 31.82 ab	41.73 ± 7.67 b	0.64 ± 0.04 ab
KF	3015.0 ± 261.63 a	28.99 ± 0.14 c	0.72 ± 0.01 a
EP3	2970.0 ± 98.99 a	45.30 ± 2.12 b	0.64 ± 0.06 abc
EP6	2825.0 ± 148.49 ab	62.36 ± 2.42 a	0.55 ± 0.01 c
EP9	2467.5 ± 67.18 b	53.56 ± 6.91 ab	0.61 ± 0.03 bc

CO: control ice cream (without kefir and elderberry pulp); KF: kefir control ice cream; EP3: kefir ice cream containing 3% elderberry pulp; EP6: kefir ice cream containing 6% elderberry pulp; EP9: kefir ice cream containing 9% elderberry pulp. Values are expressed as mean ± standard deviation (*n* = 3). Different lowercase letters within the same column indicate significant differences (*p* < 0.05).

**Table 7:** Textural properties of kefir ice creams containing different concentrations of elderberry pulp.

Sample	Hardness (g)	Stickiness (g)
CO	714.00 ± 56.60 d	−357.70 ± 35.30 b
KF	2024.00 ± 146.00 a	−389.60 ± 25.40 b
EP3	1315.70 ± 66.10 c	−272.90 ± 14.20 a
EP6	1662.20 ± 60.90 b	−286.56 ± 6.76 a
EP9	1136.80 ± 84.70 c	−279.50 ± 17.10 a

CO: control ice cream (without kefir and elderberry pulp); KF: kefir control ice cream; EP3: kefir ice cream containing 3% elderberry pulp; EP6: kefir ice cream containing 6% elderberry pulp; EP9: kefir ice cream containing 9% elderberry pulp. Values are expressed as mean ± standard deviation (*n* = 8). Different lowercase letters within the same column indicate significant differences (*p* < 0.05).

**Table 8:** Total phenolic content and antioxidant activity (FRAP) of kefir ice creams containing different concentrations of elderberry pulp.

Sample	TPC (mg GAE/100 g DM)	FRAP (µmol TE/100 g DM)
CO	388.18 ± 10.14 c	18.42 ± 0.13 d
KF	306.81 ± 11.15 d	17.54 ± 0.13 d
EP3	382.88 ± 8.85 c	28.09 ± 0.74 c
EP6	501.75 ± 2.58 b	39.04 ± 1.47 b
EP9	694.16 ± 61.17 a	49.70 ± 1.05 a

CO: control ice cream (without kefir and elderberry pulp); KF: kefir control ice cream; EP3: kefir ice cream containing 3% elderberry pulp; EP6: kefir ice cream containing 6% elderberry pulp; EP9: kefir ice cream containing 9% elderberry pulp. Values are expressed as mean ± standard deviation (*n* = 3). Different lowercase letters within the same column indicate significant differences (*p* < 0.05). TPC, total phenolic content expressed as mg gallic acid equivalents (GAE)/100 g dry matter (DM) sample; FRAP, ferric reducing antioxidant power expressed as µmol Trolox equivalents (TE)/100 g DM.

**Table 9:** Thermal properties determined by differential scanning calorimetry (DSC) of kefir ice creams containing different concentrations of elderberry pulp.

Sample	Onset Temperature (°C)	Peak Temperature (°C)	Endset Temperature (°C)	Melting Enthalpy (ΔH, J/g)
CO	−27.89	−15.06	−9.52	185.7
KF	−32.10	−21.53	−15.42	208.4
EP3	−20.56	−10.75	−8.03	161.2
EP6	−25.63	−15.28	−10.24	158.4
EP9	−25.20	−16.03	−12.34	160

CO: control ice cream (without kefir and elderberry pulp); KF: kefir control ice cream; EP3: kefir ice cream containing 3% elderberry pulp; EP6: kefir ice cream containing 6% elderberry pulp; EP9: kefir ice cream containing 9% elderberry pulp. DSC thermograms were recorded over the temperature range of −80 to 40 °C (*n* = 1). Onset temperature represents the temperature at which the endothermic melting transition begins, peak temperature corresponds to the maximum of the endothermic melting peak, and endset temperature indicates the completion of the melting transition. ΔH represents the melting enthalpy expressed as J/g.

**Table 11:** Sensory properties of kefir ice creams containing different concentrations of elderberry pulp.

Attribute	CO	KF	EP3	EP6	EP9
Appearance	8.2 ± 0.7 a	5.9 ± 2.1 c	7.2 ± 1.2 b	7.8 ± 0.7 ab	8.3 ± 1.1 a
Odor	7.0 ± 1.4 a	5.4 ± 1.9 b	6.3 ± 1.5 a	6.4 ± 1.5 a	6.5 ± 1.6 a
Flavor	7.7 ± 1.3 a	5.3 ± 2.6 b	6.9 ± 1.5 a	7.1 ± 1.7 a	7.2 ± 1.6 a
Melting in mouth	7.6 ± 1.2 a	6.0 ± 1.9 b	7.5 ± 1.4 a	7.5 ± 1.1 a	7.4 ± 1.5 a
Texture	8.0 ± 1.1 a	5.5 ± 2.2 b	7.4 ± 1.4 a	7.5 ± 1.4 a	7.5 ± 1.5 a
Graininess	8.0 ± 2.1 a	6.8 ± 2.6 b	8.0 ± 1.6 a	8.0 ± 1.8 a	7.8 ± 2.1 ab
Overall acceptability	7.8 ± 1.1 a	5.3 ± 2.1 c	6.9 ± 1.3 b	7.2 ± 1.6 ab	7.3 ± 1.3 ab

CO: control ice cream (without kefir and elderberry pulp); KF: kefir control ice cream; EP3: kefir ice cream containing 3% elderberry pulp; EP6: kefir ice cream containing 6% elderberry pulp; EP9: kefir ice cream containing 9% elderberry pulp. Values are expressed as mean ± standard deviation (*n* = 30). Sensory evaluation was performed using a 9-point hedonic scale (1 = dislike extremely; 9 = like extremely). Different lowercase letters within the same row indicate significant differences among formulations (*p* < 0.05).

## Data Availability

The original data presented in this study are openly available in Figshare at https://doi.org/10.6084/m9.figshare.33077747.
